# Exploring community support on safe motherhood: A case of Lilongwe District, Malawi

**DOI:** 10.4102/phcfm.v13i1.2907

**Published:** 2021-08-03

**Authors:** Mercy Pindani, Idesi Chilinda, Janet Botha, Genesis Chorwe-Sungani

**Affiliations:** 1 School of Nursing, Kamuzu University of Health Sciences, Blantyre, Malawi

**Keywords:** community support, safe motherhood, maternal and newborn, antenatal, intrapartum, postpartum

## Abstract

**Background:**

Malawi is grappling with a high maternal mortality of 439 per 100 000 live births. It is estimated that 80% of maternal deaths can be prevented by actively engaging the community in the country. However, community support on safe motherhood remains unknown.

**Aim:**

This study, therefore, explored community support rendered to mothers and babies during antenatal, intrapartum and postpartum periods.

**Setting:**

This study was conducted in the Lilongwe District, Malawi.

**Methods:**

This was a qualitative study that collected data from 30 village health committee members through Focus Group Discussions (FGDs). Data were analysed using thematic analysis.

**Results:**

This study found that community support on safe motherhood rendered to women varied. The following five themes emerged from FGDs data on community support on safe motherhood: antenatal support, intrapartum support, postpartum support, bylaws reinforced by chiefs in the community and safe motherhood support groups. Community members encourage pregnant women to attend antenatal care, escorted pregnant women to the hospital for delivery and assisted women to care for a child and go for postpartum checkups. Community bylaws were considered as a necessary tool for encouraging women to attend antenatal care, deliver at the health facility and attend postpartum checkups.

**Conclusion:**

This study suggests that community members play a crucial role in providing support to women and newborns during antenatal, intrapartum and postpartum periods.

## Introduction

Malawi is grappling with a high maternal mortality of 439 per 100 000 live births,^1^ and these maternal deaths occurred during the postpartum period (62.0%), antenatal (20.4%), intrapartum (9.2%) and early pregnancy from complications of abortion and ectopic pregnancy (8.4%).^2^ The World Health Organization asserts that the 24-h and the first 2-week postpartum period is the high risk of maternal deaths. The Malawi Ministry of Health recommends that women who have delivered within the health facility should receive postnatal care for 24 h before they are discharged.^1^ However, because of the lack of space in the postnatal wards, many women stay in the wards for less than the recommended 24 h. In 1996, Malawi adopted the Safe Motherhood Initiative^3^ to reduce maternal and neonatal mortality. Safe motherhood includes direct and indirect efforts to reduce deaths and disabilities resulting from pregnancy and childbirth by ensuring that every woman has access to a full range of high‐quality, affordable sexual and reproductive health services, particularly maternal and newborn care and treatment of obstetric emergencies.^4^ The Malawi Ministry of Health estimated that almost 80% of maternal deaths can be prevented in Malawi by actively engaging the community.^5^ This was in recognition that communities are a valuable source of information on why women and newborns die during antenatal, intrapartum and postpartum periods, and their participation may be critical in improving maternal health.^6^ The most common causes of maternal mortality during the postpartum period in Malawi are postpartum haemorrhage, sepsis and infection, and for the newborns or neonates, the cases are intrapartum related including birth asphyxia, prematurity and sepsis.^7^

In Malawi, some women (9%) still deliver at home.^1^ The barriers to safe deliveries are encountered at three-level decision to seek care (delay 1), reaching care (delay 2) and receiving care (delay 3).^8^ Some of the interventions that may be utilised to deal with these barriers include community transport strategy,^9^ use of maternity waiting homes^10^ and community participation.^11^ Support from the community should be highly prioritised to promote safe childbirth considering that a substantial proportion of deliveries still occur at home because of limitations in economic conditions, transportation and social infrastructure^12^ across the country. Some women prefer to deliver at home because of poor attitudes of healthcare workers (minimal communication during provision of care) as well as unsatisfactory care.^13^ In addition, women with lower levels of education are more likely to deliver at home. In Malawi, traditional leaders play a crucial role in mobilising communities for safe motherhood campaigns and encouraging women to deliver at the health facility rather than at Traditional Birth Attendants (TBA).^14^ Most husbands feel responsible for birth preparedness in the country,^15^ and they look for food and money to support their families.^16^ Some husbands also accompany their wives during antenatal and postpartum care consultations, and they are present during labour and delivery.^17^ In Ghana, some men (44.5%) accompanied their partners to seek skilled delivery services.^18^ This is important because husbands and mothers-in-law are predominant decision makers, and their roles can have a direct effect on women’s maternal health and the health of the newborn.^19^

Some community members including mothers-in-law can also act as birth companions who provide psychological and physical support to the labouring woman and provide assistance to healthcare providers.^20^ The community support for safe motherhood also includes effective collaboration between healthcare professionals and communities themselves. For instance, there is evidence that showed that communities and health facility representatives worked in partnership to investigate and respond to maternal and newborn deaths occurring in communities and health facilities in the country.^21^ In this regard, community teams succeeded in identifying maternal and newborn deaths overlooked by hospital staff, as well as deaths occurring outside the health sector.^21^

In recognition of community support on safe motherhood, the Government of Malawi prioritised the community approach by engaging communities to contribute to the reduction of maternal and neonatal deaths. This is in line with the Safe Motherhood initiative, which emphasises utilisation of antenatal care, skilled attendance at delivery and postnatal care to achieve improved birth outcomes by reducing neonatal and maternal mortality.^4^ Despite all the Government efforts, maternal and neonatal morbidity and mortality remain unacceptably high and community support on safe motherhood remains unknown in Lilongwe District. This study, therefore, explored community support rendered to mothers and babies during antenatal, intrapartum and postpartum periods.

## Methods

### Design

This was a qualitative study that explored community support rendered to mothers and babies during antenatal, intrapartum and postpartum periods.

### Setting

This study was conducted in catchment areas of one semi-rural primary health facility (Lumbadzi) and two urban health facilities (Kawale Health Centre and Bwaila Hospital) in the central region of Malawi. Kawale and Lumbadzi health centres provide labour and delivery services to approximately 13 441 and 4200 women, respectively. Bwaila serves 40 852 women of childbearing age with 8881 expected deliveries annually.^22^ Maternal and neonatal services are provided at these health facilities at no cost. In the communities, Health surveillance assistants (HSAs) in liaison with village health committee (VHC) members provide health promotion services and support to postpartum mothers and newborns during the postpartum period. The facilities were selected for this study because they provide basic emergency obstetric and newborn care (BEmONC). In addition, the facilities were working with communities that have a trained VHC that provides health promotion services on various health issues affecting the community members on a voluntary basis. Village health committee members are supposed to be oriented on various health issues to provide health-related community support.

### Sample size

The sample was purposively drawn to include VHC members from the three aforementioned communities. Thirty VHC members were recruited who were providing postpartum support to women and newborns, with 10 members from each community catchment area. The sample comprised 21 men and 9 women from each of the community catchment areas. However, the final sample size in this study was determined by data saturation. These participants were approached through the HSAs in liaison with the village headmen in the respective communities.

### Materials

A focus group discussion (FGD) guide was used to facilitate the gathering of data. We developed a FGD guide based on literature and the research question to gather data from participants. The guide was reviewed by experts in community health nursing, midwifery and mental health at the University of Malawi, Kamuzu College of Nursing. The guide contained general, open-ended questions that focussed on eliciting data on community support rendered to mothers and babies during antenatal, intrapartum and postpartum periods from VHC members. The FGD guide was tested on four community members from area 18 health centre catchment areas to refine the questions. The data from the testing of the FGD guide were excluded from the main study.

### Data collection procedure

Data were collected between November 2017 and June 2018. In this study, FGDs were conducted with the Village Health Committee to explore community support rendered to mothers and babies during antenatal, intrapartum and postpartum periods. The venue for the FGDs was purposively selected to be right in the community for easy reach of the participants. In each of the communities, FGDs were conducted in a quiet community hall that was free from disturbances and lasted approximately 60 min. The FGDs were facilitated by the researchers in vernacular language (Chichewa) to enhance better understanding and communication. Probing was carried out to elicit further details from the participants. No participant refused to participate in the discussions, and no-repeat discussions were conducted. During the discussions, participants were provided with refreshments. With participant consent, all the FGDs were audio recorded to ensure data accuracy. Furthermore, field notes were taken to enrich the audio-recorded discussions.

## Data analysis

The qualitative data from FGDs were transcribed verbatim and translated into English by the authors. The thematic analysis approach was used to analyse the data.^23^ This involved categorising concepts according to the recurrent themes that emerged across the data. The emerging themes were used to address the research questions of the study. The researcher used inductive content analysis to verify data^24^ collected during the FGDs by asking participants to confirm if ideas that were written as field notes were correct.

### Ethical considerations

Ethical approval (P.03/16/1914) to conduct this study was granted by the University of Malawi College of Medicine Research and Ethics Committee (COMREC). Written information sheets that comprised details of the study were given to participants to enhance informed decisions. Participants who were willing to participate in the study provided written informed consent. Their identities were anonymised using codes that were developed by the researchers. Only authors of this manuscript had access to participant’s data.

## Results

### Demographic characteristics of participants

The study participants comprised 30 VHC members from Bwaila (seven men and three women), Kawale (three women and seven men) and Lumbadzi (seven men and three women) health centres. There were four village headmen, one VHC chairperson and 25 VHC members.

### Community support on safe motherhood

The following five themes emerged from FGDs data on community support on safe motherhood: antenatal support, intrapartum support, postpartum support, bylaws reinforced by chiefs in the community and safe motherhood support groups. These themes have been further described.

#### Antenatal support

The community members revealed that they provided various forms of support to pregnant women during the antenatal period. They encouraged pregnant women to attend antenatal care as soon as they realised that they were pregnant for proper monitoring of the pregnancy. Pregnant women in the community also influenced each other on issues of antenatal care, and they went together to antenatal clinics. The participants further disclosed that most of the elderly women in their communities took time to teach first-time mothers on how to handle pregnancy as part of their tradition. Similarly, when a woman was pregnant, community members assisted her with household chores such as farming, fetching water and firewood, going to the maize mill and cooking. Other community members were also sensitised on the dangers of engaging pregnant women in excessive manual work that could trigger premature labour. As such, they were encouraged to ensure that pregnant women were only engaged in light duties and exercises. Birth preparedness came out as another important element of antenatal support. The participants revealed that they encouraged pregnant women to identify appropriate support persons, transport arrangement and resources that would be needed to support the arrival of the baby such as plastic paper, thread, baby clothes, razor blade and wrappers for the woman. According to the participants, birth preparedness was necessary for the pregnant woman to be ably assisted during the emergency delivery.

‘People in the community […] encourage each other to be attending antenatal clinics when pregnant. For instance when two or three pregnant women meet, they ask each other when last each one of them went to the hospital for antenatal care, or if they had started at all. Then they set dates when they can go together …’ (Participant 3, Kawale FGD)

‘People in the community sensitize one another on dangers of engaging a pregnant woman in heavy or tiresome work or household chores. For instance if a man is seen overworking his pregnant wife such as letting her work at the garden from morning up to afternoon, he is told to stop such behaviours.’ (Participant 1, Kawale FGD)

‘When the woman is pregnant she is also supposed to start preparing for things to carry with her to the hospital when she goes in labour such as plastic paper, thread, wrappers and razor blade, because when labour starts, it becomes difficult for her to start packing all the necessary things […] Because sometimes you can even deliver on your way to the hospital, so if you did not prepare, those people who have escorted you find it difficult to assist you but if you have everything ready it is easy they can assist you and then proceed to the hospital.’ (Participant 5, Bwaila FGD)

#### Intrapartum support

The study found that intrapartum support was another important component of community support towards safe motherhood. Community members revealed that they encouraged pregnant women to deliver at the hospital where they could be assisted by skilled health workers. They emphasised that they discouraged pregnant women from delivering at TBAs where they could not be assisted in cases of emergencies or complications. They further reported that when a pregnant woman went into labour, they escorted her to the hospital for delivery. At the hospital, they kept the pregnant woman company until she delivered her baby. Soon after the delivery, they prepared porridge for the woman to eat to regain her strength:

‘When a pregnant woman is due for delivery, we escort her to the hospital and support her at the hospital until she delivers her baby.’ (Participant 3, Lumbadzi FGD)

‘When it is during the night and the woman goes into labour, and when her husband is not around, we call each other as a group of men and women in the village to take her to the hospital. Sometimes we call for a car or a bicycle to take her to the hospital. When a woman has just delivered a baby, her neighbours prepare porridge for her to eat so that she regains her energy. After this, they continue giving her other foods to eat.’ (Participant 4, Bwaila FGD)

#### Postpartum support

Participants indicated that they provided various forms of support to a woman who had delivered including her neonate. Immediately after discharge from the hospital, they took the woman and her neonate home, where they stayed with them until the mother completely regained her strength to support herself. During this time, the community members taught the mother how to take care of the baby and helped her with household chores such as smearing the floor, cooking, laundry and taking care of other children. These activities were carried out by the family relatives, neighbours and other members of the community even when they were not related to the family. The participants further reported that community members monitored the growth of the neonate to detect whether the child was growing well or was malnourished. When they detected that the neonate’s growth was faltering, they encouraged the mother to go to the hospital for further management. In a situation where the woman had lost her baby, members explained that women from the community were the ones who did the funeral undertakings to assist the woman go through the grieving process:

‘When the woman is discharged from hospital, we take her home. At home, we usually do the household chores for her until the time she regains strength and she is able to support herself. Community members who come to see the baby also monitor the growth of the neonate. If they notice, that the neonate is not growing healthy, they encourage the mother to go to the hospital to seek medical attentions.’ (Participant 6, Lumbadzi FGD)

‘Community members also help the postpartum mothers with smearing the floor, cooking as well as looking after and feeding the other children since the mother is busy with the newborn. The community members also bring gifts like soap and clothes for the newborn and assist the postpartum mother with her laundry.’ (Participant 5, Bwaila FGD)

#### Initiatives and bylaws by chiefs in the community

The participants revealed that the antenatal, intrapartum and postpartum support that the community rendered towards safe motherhood initiatives was further reinforced by the bylaws that were set and implemented by the chiefs in their respective communities. All the FGDs revealed that the chiefs in their communities had set bylaws that compelled women to attend antenatal care and delivered the baby at the health facility. Furthermore, these bylaws required women to attend postpartum checkups at the health facility. Male involvement in issues of safe motherhood was also covered in the bylaws. Participants disclosed that if community members did not adhere to the bylaws, they were forced to pay a goat or money equivalent to the price of a goat:

‘Some chiefs have put an order that every woman should deliver at the hospital and also that husbands should accompany their wives to the antenatal clinic. For this rule to work, the chiefs have ordered a punishment in terms of money, or goat. These are things that the community members can hardly afford as such they are forced to go to the hospital because they do not have a goat or money to pay. Chiefs call for meetings to make people aware of such by-laws and the consequences of not following them.’ (Participant 2, Kawale FGD)

#### Safe motherhood support groups in the community

Safe motherhood support groups in the community were identified as an important element of community support. The FGDs yielded three sub-categories under this theme namely: availability of support groups, need to introduce support groups and expected role of support groups. Participants from all three health facilities divulged that there were no support groups specifically for safe motherhood activities. However, it was revealed that at one facility, there was a support group that dealt with orphans. The participants were of the view that the introduction of support groups for safe motherhood in all the health facilities was necessary. They recommended that once introduced the support groups ought to have clear protocols and guidelines on how to operate to ensure that the groups focussed on safe motherhood issuers other than general sanitation issues that most community health workers focussed on. Despite showing the need for introducing support groups on safe motherhood, the participants were not knowledgeable of the expected roles of the support groups. They, therefore, indicated the need for training and capacity building when the support groups were being introduced:

‘I have never heard about this group in our area, what you are asking about is news to us. We only know about the support group working with orphans and is supported by the SOS children’s village but not looking at maternal and neonatal health.’ (Participant 1, Kawale FGD)

‘There is no any special group that focus on issues of safe motherhood only. The village health committee just combines everything as long as they are health issues. To properly assist the community on safe motherhood, there is need to establish a special group with clear protocols and guidelines on how to operate.’ (Participant 1, Lumbadzi FGD)

## Discussion

The study revealed various forms of support that community members provided during the antenatal, perinatal and postnatal periods apart from the use of bylaws to promote maternal and neonatal health. The findings of this study showed that community members encouraged pregnant women to attend antenatal care as soon as they realised that they were pregnant as well as taught first-time mothers on how to handle pregnancy, escorted pregnant women to the hospital for delivery and at times stayed with them until discharge and taught women how to take care of the baby and helped with household chores. Community members would provide maternal and neonatal support even when there was no direct relationship. Similar sentiments have been reported by the WHO that asserts the importance of community support in accompanying the woman to the hospital for antenatal, labour and postpartum care.^25^ They further recommend the need for community members to recognise and respond to danger signs during pregnancy, labour and postpartum.^25^ Furthermore, Lassi, Kumar^26^ asserted the importance of community support through home visitations to a postpartum woman and neonate. They report that community home visitations may lead to increased breastfeeding practices, early detection of complications to the mother and neonate as well as timely referral to a health facility. It seems the culture of extended family arrangement that was common in African countries including Malawi, compelled members to take responsibility towards fellow community members when they were pregnant, in labour and during the postpartum period. Masala-Chokwe and Ramukumba^27^ noted that this kind of support was essential especially to first-time mothers as they mostly lacked knowledge and skills of maternal and neonatal issues. These findings are in keeping with the WHO’s Alma-Ata Declaration that stressed on involving community members in health initiatives as an inseparable component of health programmes.^28^

Interestingly, support towards maternal and neonatal health from families and other community members has also been reported by other studies in Africa. Mbekenga, Christensson^29^ found that postnatal women including first-time mothers in Tanzania significantly relied on family members and neighbours, in any instances of uncertainty on how to handle things during the postpartum period. Likewise, in Nigeria, some rural communities were found to have some form of emergency transport arrangements in place to assist women when they experienced a maternal obstetric emergency.^30^ However, community support towards maternal and neonatal care needed to be considered with caution, as most of the retrogressive cultural beliefs and practices, uncovered in this study, were perpetuated by the same community members. Nevertheless, the involvement of the community was vital in improving maternal and neonatal health.

The study further found that community bylaws passed by local leaders played an important role in promoting maternal and neonatal health. In Malawi, some people believe that instituting bylaws to prevent traditions posing a risk to pregnant women may improve maternal and newborn health.^21^ The Malawi Ministry of Health gave the mandate to local leaders to develop and enforce bylaws on issues of reproductive health to prevent traditions posing a risk to pregnant women.^14,31^ The traditional leaders may pass local bylaws that provide for all women in the community to deliver at a health facility and that those who disobey shall be penalised or fined a goat or a chicken.^32^ Nonetheless, these bylaws are not legally binding.^14^ Literature suggests that women and their families obey bylaws to deliver in the facility out of respect for the traditional leader, which is entrenched in Malawian culture.^14^ Conversely, fear of penalties or fines prescribed by bylaws compel women to attend antenatal care, deliver at the health facility and attended postpartum checkups on time.^14^ Although this study did not assess the effectiveness of community bylaws, studies conducted in Swaziland and Zimbabwe found bylaws to be useful and acceptable supporting tools for changing community norms.^33^ Conversely, a study conducted by Lodenstein and colleagues found that bylaws that oblige women to attend antenatal care are problematic and discriminate against women in the definition and application of sanctions in the northern part of Malawi.^34^ Consequently, the sanctions may negatively impact on women’s reproductive health, rights and equity.^34^

The various forms of community support unveiled by this study were, however, not complemented by any support group that specifically spearheaded maternal and neonatal health issues although there was a strongly expressed need for the establishment of such groups. In Zambia, community-based volunteer groups, known as safe motherhood action groups (SMAGs), were found to be very instrumental in increasing utilisation of institutional deliveries.^35^ The mother support groups instituted in communities to support maternal and child health in Sri Lanka were also found to be effective in promoting positive maternal and neonatal health practices.^36^ Consistently, Gai Tobe, Islam^12^ reported that community women’s groups who practice participatory learning and action during pregnancy, delivery and postpartum significantly improve the utilisation of antenatal, delivery and postnatal care services and a reduction in maternal and neonatal mortality. These studies have rendered empirical support on the need for the establishment of community support groups and their effectiveness in promoting maternal and neonatal health.

## Conclusion

This study suggests that community members provide support to women and newborns during antenatal, intrapartum and postpartum periods. Community members encourage pregnant women to attend antenatal care, escorted pregnant women to the hospital for delivery and assisted women to care for a child and go for postpartum checkups. Community bylaws were considered as a necessary tool for encouraging women to attend antenatal care, deliver at the health facility and attend postpartum checkups. Support groups focussing on maternal and neonatal health were lacking in the community. However, there was willingness from community members to establish safe motherhood support groups.

## Implications for practice

Considering the high maternal and neonatal mortality rate prevalent in Malawi and that community members play a crucial role in caring for women during antenatal, intrapartum and postpartum periods, there is a need to enhance measures for strengthening community support on safe motherhood in health facilities across the country. It is also necessary to conduct implementation science research to identify barriers, enablers and come up with an effective implementation strategy for community support on safe motherhood in the local setting.
